# The Value of an Incomplete Degree: Heterogeneity in the Labor Market Benefits of College Non-Completion

**DOI:** 10.1080/00221546.2019.1653122

**Published:** 2019-08-20

**Authors:** Matt S. Giani, Paul Attewell, David Walling

**Affiliations:** a Office of Strategy and Policy, The University of Texas at Austin, Austin, Texas, USA; b Graduate Center, City University of New York, New York, New York, USA; c Texas Advanced Computing Center, The University of Texas at Austin, Austin, Texas, USA

**Keywords:** Degree completion, incomplete degree, college dropout, post-college earnings

## Abstract

Many undergraduates leave college without completing a degree or credential. Some researchers characterize this as a waste of the student’s time because (they assert) college short of a degree does not yield any advantage in the labor market. Using data for an entire cohort of students graduating high school in Texas in one year, we compare the employment and earnings years later of those who do not go beyond high school with those who enter college but do not complete a credential. Using techniques that address selection bias, we find that students with “some college” are considerably more likely to be employed fifteen years after high school graduation and tend to earn significantly more than their counterparts who do not go to college. These benefits are found across student subgroups, with low-income students, women, and students of color generally experiencing the greatest improvements in labor outcomes from college attendance. While college dropouts do not fare as well as college graduates, incomplete college nevertheless functions for many as a stepping-stone into a better labor market position.

Enrollment in American higher education grew from 13.2 million undergraduates in 2000 to 17 million in 2016 (McFarland et al., 2017). The proportion of 25 to 29 year-olds with at least an Associate’s degree grew from 38% to 46% over the same period, and the proportion with a baccalaureate or higher credential increased from 29% to 36% (McFarland et al., 2017, p. 43).

Despite these upward trends, commentators point to the fact that many undergraduates do not complete their course of study. For the national cohort beginning college in 2010, 37% of the students entering four-year public colleges and 61% at public two-year community colleges had not graduated with a credential within six years.^1^ In popular parlance, many undergraduates, including the majority of community college entrants, “stop out” or “drop out” of college before completing a credential. In government surveys, these individuals are usually classified as “some college” (i.e., attending college short of a credential).

This situation has been labeled a crisis in higher education by some policy makers and advocacy organizations who view unfinished degrees as a problem for the economy as a whole, as well as for the individuals directly involved (Complete College America, 2011; Hess, Schneider, Karey, & Kelly, 2009; Schneider & Yin, 2012). In particular, there is a narrative that students who leave college before graduation and therefore lack a credential have largely wasted their own and taxpayers’ time and resources. Concerns about incomplete degrees have led many states to set ambitious postsecondary attainment goals (HCM Strategists, 2016; Lumina Foundation, 2017) and have contributed to the expansion of policies whereby postsecondary institutions are funded for graduates produced rather than students enrolled (Friedel, Thornton, D’Amico, & Katsinas, 2013; Jones, 2014; National Conference of State Legislatures, 2014; Tandberg & Hillman, 2014).

The importance of degree attainment is difficult to dispute given the large and growing earnings premiums of college graduates (Baum, 2014), the equalizing effects of earning a degree (Giani, 2016; Hout, 1988; Torche, 2011), and the diverse non-monetary benefits to college attainment (Hout, 2012; Pascarella & Terenzini, 2005). However, colleges and universities may respond to the increasing emphasis on completion in one of two ways. One approach, with few critics, is to develop new strategies for supporting students and ensuring they complete their degree. A second approach is to limit college access for those who are less likely to complete a degree. Indeed, college leaders in states implementing outcomes-based funding have told researchers explicitly that they have considered or taken practical steps to limit access to students who are less likely to complete (Dougherty et al., 2014). This approach may indeed lead to higher degree conferral rates, but whether it is a wise (and ethical) response hinges on the benefits accrued from college attendance short of completion. This question is of particular importance given that low-income students and students of color may be disproportionately excluded from higher education if students from under-resourced schools and communities are viewed as having lower odds of success.

A number of studies have investigated the labor market benefits of college attendance and found evidence that even non-completers have higher earnings and better employment rates than high school graduates who never attended college (Jacobson, LaLonde, & Sullivan, 2005; Jepsen, Troske, & Coomes, 2014; Kane & Rouse, 1995, 1999; Marcotte, Bailey, Borkoski, & Kienzl, 2005). However, others have found that the outcomes of college non-completers are no different than high school graduates (Carneiro, Heckman, & Vytlacil, 2011; Holzer & Baum, 2017; Rosenbaum, Ahearn, Becker, & Rosenbaum, 2015).

Given this ambiguity in the literature and the importance of understanding the relationship between college non-completion and labor market outcomes, the present study contributes in four ways. First, we study a statewide cohort of students who graduated high school in Texas in the year 2000, allowing us to directly compare college non-completers to non-college-goers, in contrast to the majority of studies that use cohorts of beginning postsecondary students to estimate the impact of college non-completion. Second, we examine this cohort’s labor market outcomes using Unemployment Insurance (UI) data in 2015, fifteen years after high school graduation. This provides sufficient time to earn a credential (or not), and yields longer term estimates of labor market outcomes. Third, we use a technique called Augmented Inverse Probability Weighting (AIPW) that reduces selection bias and provides more accurate estimates of the impact of college attendance short of a credential. We also explore alternative modeling strategies and find that our results are generally robust to alternative specifications. Finally, the sample size of more than 200,000 students allows us to identify sources of heterogeneity in the relationship between college non-completion and labor market outcomes based on economic background, race/ethnicity, gender, and whether students attended a 2-year or 4-year college.

Our analyses provide additional evidence for the economic value of an incomplete degree. We find that, for both two-year and four-year college entrants, college attendance short of a degree or other credential does “pay off” in terms of a much higher likelihood of being employed fifteen years later and in terms of significantly higher earnings, compared to classmates who did not go beyond high school graduation. This payoff is evident for students who persist in college for a relatively short time, as well as for those who come closer to finishing a degree. The employment benefit from having “some college” short of a degree is even greater for historically under-represented groups, such as economically disadvantaged students, women, and students of color.

In the conclusion, we discuss the implications of these findings. While strongly indorsing the importance of a credential as a goal for all college students, we argue that the current emphasis on completion should not come at the expense of college access, particularly for historically marginalized student populations. We further argue for a conceptual shift away from the notion of “the college dropout” as failure or wasted effort and toward an appreciation of the practical utility of “some college” for many students. Our findings suggest that, on average, high school graduates who attend college without completing a credential are considerably better off in terms of employment and earnings^2^ than they would have been, had they ended their education at high school graduation. We also consider several mechanisms that explain why college short of a credential can still lead to positive employment outcomes.

## Human capital, signaling, and the economic effects of college non-completion

One of the ironies of the current policy preoccupation with college completion is its misalignment with the theoretical framework that is perhaps most responsible for the expansion of — and increased government investment in — higher education, namely human capital theory. Put simply, human capital theory posits that education provides students and workers with knowledge and skills that have economic value and can be exchanged for better pay and employment prospects in the labor market (Becker, 1962, 1975). Although establishing the causal relationship between education and earnings is difficult given the inherent self-selection bias in who attends college, the most rigorous evidence available suggests a causal link between increased educational attainment and improved labor outcomes (Card, 1999).

Mincer (1974) is credited with pioneering the most widely used statistical representation of this relationship by linearly relating years of education with log-earnings, controlling for years of work experience and other time-invariant worker characteristics (race/ethnicity, sex, measures of ability, etc.). The coefficient of the years of education variable is often interpreted as the “return to schooling,” although this interpretation has been criticized and the “average growth rate of earnings per additional year of education” is likely more accurate (Heckman, Lochner, & Todd, 2006). Regardless, whether individuals received a postsecondary credential was not part of the original formulations of human capital theory or the Mincerian earnings equation; the knowledge and skills gained from higher education, proxied by years in school, were viewed as more valuable than the credentials provided by educational institutions.

Despite this tradition, and early work that found limited discontinuities in the “returns to schooling” around years of education that corresponded to when students receive credentials (Layard & Psacharopolous, 1974), more recent research has tended to emphasize the importance of earning a college credential. This emphasis has emerged from research showing that workers with credentials earn significantly more than similar workers with equivalents amounts and types of education but no degree or certificate (Hungerford & Solon, 1987). This finding has been termed the “sheepskin effect” in the economics of education literature to describe the additional benefits that appear to be provided by a credential above and beyond the “human capital” benefits of the knowledge and skills gained from one’s educational experience.

Michael Spence (1974) won the Nobel Prize in economics in part for his asymmetric information theory of the signaling function of education, in which employers read educational credentials as indicative of the likely quality of job seekers because they lack more direct knowledge of an applicant’s workplace abilities. Spence had in mind the positive signal associated with a *completed* educational credential. However, Spence’s logic might also be relevant for less-educated young workers who are seeking entry-level jobs immediately after stopping out of college. Employers may view young adults with some college as more appealing to hire than high school graduates with no college exposure. In an era where 70% of young people start college immediately after graduating high school and many more make the transition in the years following (Bureau of Labor Statistics, 2017; Horn, Cataldi, & Sikora, 2005), those job applicants who have not at least tried college may encounter suspicion on the part of employers, for lacking drive, ambition or cognitive ability.

Similar signaling may occur when undergraduates apply for jobs or internships while still enrolled in college. The majority of today’s undergraduates (62%) work for pay while they are also enrolled in college (Carnevale, Smith, Melton, & Price, 2015; Kena et al., 2016). For such students, paid jobs and internships undertaken while enrolled in college may be stepping-stones to post-college employment. Employers may view students who are “working their way” through college as more meritorious. High school graduates who never attend college may lack these “foot in the door” work opportunities.

Although signaling theory might explain both the sheepskin effect and the positive relationship between college non-completion and labor outcomes, it is not immediately apparent how it might explain a complete lack of labor market benefits for students who do not receive the credential. Would failure to earn a credential nullify the potential labor market benefits provided by gains in knowledge and skills? One possibility would be that the specific courses and credits students earned in college did not provide them with the knowledge and skills valued by the labor market. However, an extension of signaling theory would state that, given the growing emphasis on college completion, dropping out of college might indeed be a powerful enough negative signal to outweigh any benefits provided by college attendance short of a degree.

This underscores an important but often implied point — signals are heavily contextual. A bachelor’s degree in a specific field from a specific college or university could be a positive signal for certain job opportunities and a negative signal for others. Similarly, college attendance without completion could be a positive signal for some job opportunities and a negative signal for others. More precisely, the average economic benefits provided by a given level of education or credential are proportional to the relative supply and demand of workers with those skills in that particular segment of the labor market.

Just as there may be heterogeneity in the returns to schooling and the benefits of college completion by the field of education and employment, there may exist heterogeneity in who benefits the most from higher education and — for our purposes — from college attendance short of completion. As Brand and Xie (2010) note, researchers have historically assumed the existence of *positive selection*, or students who are most likely to benefit from higher education also being most likely to attend. In contrast, Brand and Xie provide evidence of *negative selection*, or students least likely to attend college deriving the most benefit from doing so. As they state:

In the absence of a college degree, low [college-going] propensity men and women have limited human, cultural, and social capital and hence particularly limited labor market prospects. By contrast, in the absence of a college degree, individuals from more advantaged social backgrounds can still rely on their superior resources and abilities. (p. 293)

Brand and Xie estimate the economic benefits of college completion rather than non-completion and produce estimates for different propensity score strata rather than demographic groups. We extend this line of reasoning to posit that college attendance short of completion may be a more positive signal for individuals from groups historically disadvantaged in the labor market compared to their more privileged peers. The following section further reviews the empirical studies that have examined the relationship between college non-completion and labor outcomes overall, as well as potential heterogeneity in the returns to college non-completion.

## Prior empirical studies on the labor market benefits of college non-completion

As alluded to above, the empirical literature on the benefits of college attendance sans attainment is quite mixed. Reviews by both Kane and Rouse (1999) and Belfield and Bailey (2017) report significant earnings payoffs to an incomplete degree even at community colleges across a variety of studies and state contexts. Belfield and Bailey (2017, p. 10) conclude: “ … there are positive returns to human capital accumulation in college even when a student does not complete an award … The association is broadly linear.” Kane and Rouse (1995, 1999) similarly concluded that the returns to credits for non-completers at both 2-year and 4-year colleges are quite similar and in the range of 5–8%.

Backes, Holzer, and Velez (2014, p. 16) analyze administrative data for Florida, noting: “… there are also returns, on average, to attending a program and earning credits, even if that program is not completed. In addition, there are larger returns (relative to those with no postsecondary enrollment) to accumulating more credits.” This is congruent with Jacobson et al.’s (2005) finding that each academic year of community college attendance raises the long-term earnings of men by nine percent and women by 13 percent.

On the side more skeptical of the benefits of college attendance without attainment, one influential statement, under the egis of the William T. Grant Foundation, is Rosenbaum et al.’s (2015) study which concludes: “… The dominant finding presented here is that the most frequent outcome for community college students is ‘some college’—no credentials, no earnings payoff, and potentially substantial debts” (p. 14). This claim—equating some college to no earnings payoff—is based on their analyses of the Educational Longitudinal Survey (ELS). A similar conclusion is offered by Holzer and Baum (2017, p. 1) who write: “Many students leave school [i.e. college] without any certificate or degree. They have lost valuable time and frequently have student debt to repay, but they have not managed to measurably improve their prospects.” Carneiro et al. (2011) focus on “marginal” students who are induced to attend college by a policy change promoting access and find the estimated returns to college for marginal students are lower than estimates for the average college-goer. Their conclusion is similarly emphatic: “This policy induces students who should not attend college to attend it” (Carneiro et al., 2011, p. 2755).

Scott-Clayton and Wen (2018) report a more complex picture based on analyses of the National Longitudinal Survey of Youth for 1997 (NLSY97). Individuals with “some college” at a four-year institution do receive a substantial earnings payoff, but the earnings of non-completers at community colleges are no different from those of high school graduates who never attended college. Marcotte et al. (2005) report that each additional year of enrollment at a community college improves yearly salary (and less so hourly wages). However, their estimates of the effects of community college attendance on earnings were no longer significant once high-school fixed-effects had been controlled for.

In summary, previous studies disagree with one another on the basic question of whether students who attend college without earning a credential gain in terms of enhanced likelihood of employment and/or higher earnings post-college. The most likely explanation for these conflicting results is the diversity of samples, datasets, and methods used in this line of research. The following section examines this concept further by reviewing literature on heterogeneous returns to education.

### Variation in the effects of college non-completion

The majority of studies estimating the returns to postsecondary education, whether for students who attain a credential or for non-completers, focus on the average returns to college. However, it is important to consider heterogeneity in the returns to college-going. The literature suggests three sources of variation are most important in moderating the relationship between college attendance and labor outcomes: type of institution attended, type of credits earned, and type of student enrolling.

Although studies have investigated variation in the returns to college attendance by the type of institution students enrolled in, a clear pattern has yet to emerge. Some studies have found attending a 4-year college provides an earnings boost while community college attendance does not (Marcotte et al., 2005; Scott-Clayton & Wen, 2018), some have found that the earnings payoff is roughly similar for attending a 2-year or 4-year college (Kane & Rouse, 1995, 1999), and some have found benefits of community college attendance even larger than the estimates commonly found for 4-year colleges (Jacobson et al., 2005). These conflicting findings suggest more research is needed in this area.

Another potential source of heterogeneity in the returns to college non-completion is the type of credits students earn in college. Although there appears to be less research in this vein compared to research on how the returns to college credentials vary by major (Arcidiacano, 2004; Walker & Zhu, 2011), there is some evidence to suggest that credits more directly applicable to workforce needs, such as career and technical education credits earned at a community college, provide greater labor market benefits then general academic credits (Bahr, 2016; Grubb, 1992, 1993).

Finally, the returns to college attendance without attainment could vary by the types of students attending college. Carneiro et al. (2011) concluded that the benefits of college attendance for marginal students were essentially nil. They used this finding to contend that expanding access to college likely induces students to attend who should in fact forgo college.

In contrast, Brand and Xie (2010) found larger returns to education for students with the lowest estimated propensity to go to college. This finding was similar to Card’s (1993) pioneering study using geographic proximity to a college as an instrument to model the effect of college-going, in which he found that the earnings benefits of college attendance for men were concentrated among students with poorly-educated parents—those students least likely to attend college.

Examining how the benefits of college vary by demographics is particularly important given both demographic disparities in access to college and the fact that a number of influential studies included samples of only men (Card, 1993) or white men (Carneiro et al., 2011). Other studies have estimated how the economic benefits of college vary by gender, and the majority provide evidence to support the hypothesis that women receive greater benefit from college than men (Bahr et al., 2015; Dadgar & Trimble, 2015; Jacobson et al., 2005; Jaggars & Xu, 2016; Jepsen et al., 2014; Minaya & Scott-Clayton, 2017).

Less research has examined how the returns to college-going vary by race/ethnicity and socioeconomic origins. Monks (2000) reported evidence that the returns to college were larger for non-whites compared to whites, particularly for women. For most students, Dale and Krueger (2011) found limited effects of higher levels of institutional selectivity on earnings; however, for Black, Hispanic, and first-generation students, the returns to college selectivity were large and significant. Zimmerman (2014) exploited a discontinuity in GPAs used for admissions to identify the effect of enrollment at a 4-year college on marginal students’ earnings. Although college-going provided limited benefit to higher-income students, the benefits were large and significant for low-income, Black, and Hispanic students. While these studies suggest the existence of heterogeneity in the returns to college based on race, class, and sex, their focus was not on the returns to college non-completion specifically.

These latter results suggest students from historically underrepresented groups may benefit even more from college-going than students from more privileged backgrounds, but the general lack of research in this area prevents firm conclusions from being drawn. In what follows we will address two issues: first, determining whether there are benefits in post-college employment and earnings for college attendance short of a degree, and secondly, whether there is heterogeneity in those benefits across race/ethnicity, gender, economic background, and the type of institution attended.

## Data and methods

### Data source and sample

To address these questions, we use administrative data from the Texas Education Research Center (TERC) at the University of Texas at Austin. TERC manages Texas’ statewide longitudinal student data system that links K-12 data collected by the Texas Education Agency (TEA) to postsecondary data collected by the Texas Higher Education Coordinating Board (THECB) and to employment data collected by the Texas Workforce Commission (TWC). Every student who attends a public school in Texas between early elementary and high school graduation is included in the data repository and is trackable through postsecondary education and into the workforce with a unique identification number, as long as the student remains in Texas. This data repository has been used to study students’ pathways through the K12 and postsecondary education system and into the labor force (Andrews, Li, & Lovenheim, 2014, 2016). However, to the authors’ knowledge the relationship between college non-completion and labor market outcomes has not been explored using this data source.

The population used here includes all persons who graduated from a Texas public high school in the year 2000 (*n* = 207,332). Of this population, 147,650 (71.2%) attended an in-state community or technical college, or public 4-year college, or private 4-year college, or in-state for-profit college: 102,590 (49.7%) enrolled in a public 2-year college, 38,396 (18.5%) enrolled in a public 4-year college, 3,473 (1.7%) in a private 4-year college, and 3,191 (1.5%) in a for-profit school (the issue of students who left Texas is discussed in a separate section below). Separate models are fit to the 2-year and 4-year college sub-samples, as described further below.

Although THECB does collect data on whether students enroll in in-state private 4-year colleges and for-profit colleges, no data on credits attempted or earned is collected for these postsecondary sectors. Thus, for the small subset of analyses below that estimate the impact of credits on earnings, the college-going group is restricted to students who enrolled in a *public* 2-year or 4-year college. For all other analyses, college goers who attend either public or private colleges are included.

To address the first research question about the payoff to incomplete or some college, we restricted analyses to two groups: (1) high school graduates of spring 2000 who never attended any Texas post-secondary institution through the end of calendar year 2014 (the comparison group) and (2) persons who did attend a college within the same time period but did not complete any credential (the treatment group). Table 1 shows the highest credential received by Texas students in the 2000 cohort.

**Table 1.** Highest postsecondary credential/credits earned by 2014.

	Frequency	Percent
No College	61,486	29.7
No Credential — 1–12 Credits	11,776	5.7
No Credential — 13–60 Credits	32,494	15.7
No Credential — 61+ Credits	24,296	11.7
Occupational Skill Award (OSA)	312	.2
Short certificate	5713	2.8
Long certificate	623	.3
Academic associates	6545	3.2
Applied associates	5411	2.6
Bachelors of applied technology	805	.4
Bachelors	45,682	22.0
Graduate	12,189	5.9
Total	207,332	100.0

Quarterly earnings data for year 2015 come from administrative records from the unemployment insurance (UI) system overseen by the TWC. With exceptions described below^3^, employers in Texas are required by law to report earnings data for their employees to this agency (Aspen, 2014).

### Variables

Three dependent variables represent post-college outcomes: (1) whether a person had any non-zero earnings in any quarter of 2015 according to the UI system, a dichotomy we term “partial employment;” (2) whether a person had non-zero earnings for all four quarters in 2015, a dichotomous variable we term “full employment;” and (3) a person’s total reported earnings for 2015, which we top-coded at the 99th percentile of earnings for our population ($214,554.30) and then took the natural logarithm. We label this variable “earnings.” However, we also ran models with different specifications of earnings, as discussed in a section on robustness below. In addition, the findings on partial employment and full employment were nearly identical. We therefore present the findings on full employment, but the results on partial employment are available upon request to the corresponding author.

The main independent variable (or treatment variable) is a zero/one dummy variable where a value of one indicates that the individual did attend college without earning a credential and zero indicates that the individual graduated high school but did not attend an in-state college. In some models we use a categorical version of the credits variable that places students into one of four categories based on the college credits they earned: (1) no credits/college attendance, (2) 1–12 credits, (3) 13–60 credits, and (4) 61+ credits.

The Texas educational database contains the following demographic variables used in the analyses: race/ethnicity (American Indian, Asian, Black, Hispanic, and non-Hispanic White); gender; and economic disadvantage, a four-level categorical variable for students who: (1) are not disadvantaged, (2) qualify for free lunch under the Federal government program, (3) qualify for reduced-price lunch, (4) are otherwise economically disadvantaged. We collapsed this variable into a dichotomous variable by combining the latter three categories into the disadvantaged subgroup.

Several variables represent students’ high school academic skills and preparation. In this time period, Texas required high school graduates to take standardized tests called the Texas Assessment of Academic Skills (TAAS) that assessed skills in math and reading. We use students’ percentile scores (1–99%) on the math and reading exams administered in 10th grade both as a statistical control for academic achievement in models predicting employment and earnings, and also when modeling selection into treatment. Additional academic variables include the number of advanced courses completed in high school, the number of dual credit courses completed in high school, and the total number of high school credits students earned. Finally, a dichotomous variable was used indicating whether students were identified as gifted and talented by their public school. Descriptive statistics for these variables are provided in Appendix 1.

Because the characteristics of the high school from which a student graduates may influence both their likelihood of attending college and their earnings potential, we controlled for high school characteristics in predictive models. For OLS and logistic models we employed high-school fixed-effects: a set of dummy variables presenting the high school from which each student graduated. In selection models (discussed below) high school background was represented instead by five school-level characteristics: (1) percent economically disadvantaged in the school, (2) percent female (3) percent under-represented minority (Black, Hispanic, and Native American) (4) percent of high school graduates who attended any postsecondary education the year after high school, and (5) percent of high school graduates who enrolled in a 4-year college after high school.

#### Modeling strategy

The simplest strategy for estimating a difference in outcomes between incomplete-college and high-school- only students involves either a logistic regression (predicting dichotomous employment fifteen years after high school graduation) or an OLS regression when predicting log earnings in that year. In both cases, the regression contains a ‘treatment’ variable (i.e., attending college short of a degree) in addition to control variables for the student’s demographic and family background, and for each student’s academic achievement in high school. This was the approach taken by several of the studies reviewed earlier, and for comparability we initially ran similar models.

However, regression models, even when they contain covariates representing potential confounders, may not adequately correct for selection into treatment on observed variables (Winship & Morgan, 1999). Several strategies, known as “Counterfactual Models,” “Potential Outcome Models,” or “Treatment Effect Models” estimate the effect of a single “treatment variable” on an outcome, while taking into account selection into treatment. We employ one of these, called Augmented Inverse Probability Weighting (AIPW), to address selection effects. We use the procedure called “teffects aipw” in Stata13 for these estimations (StataCorp, 2013).

This method first estimates a model to predict the likelihood of experiencing the treatment (in this case, predicting who attended college short of a degree) using as predictors each student’s socio-demographic variables, variables representing their academic preparation and achievement in high school, and five high-school characteristics. For each person, this first-stage model yields a predicted probability of receiving the treatment, often referred to as the propensity score (Rosenbaum & Rubin, 1983). In the more commonly used technique of propensity score matching, treatment and control cases are matched to each other based on their propensity score, and unmatched cases are excluded from the analysis. One way to understand propensity score matching is that each case receives a weight based on whether they are included in the matched sample; unmatched cases receive a weight of zero and do not contribute to the estimation, whereas matched cases receive a weight of one and contribute fully to the model. The difference in the means between the matched groups constitutes the average treatment effect (ATE).

With Inverse Probability Weighting (IPW), the inverse of the propensity score functions as a case weight when estimating a second-stage model, now predicting the outcome (employment or log earnings) with the predictors consisting of the treatment dummy indicating some college, plus the full range of available socio-demographic, educational, and high-school-level covariates. Concretely, the higher the propensity score, the less that case contributes to the estimation because s/he is very different than the average student in the control group on observed characteristics (higher achieving, higher SES, etc.). The use of inverse probability weights acts to reduce imbalances between the treated and untreated cases on measured covariates, adjusting for selection. Treatment models of this type are common in medical and economic research but are relatively new to other social sciences. See Xie, Brand, and Jann (2012) for a comprehensive discussion.

AIPW is a modification of this approach that enhances robustness and efficiency of estimation (Rubin & van der Laan, 2008; Tan, 2010). After computing inverse-probability weights predicting treatment status, an AIPW analysis estimates separate regressions for the treatment and control groups to obtain estimates of the group-specific outcomes for each. Differences in the weighted means of each treatment level regression provide the Average Treatment Effect (ATE). Simulations by Glynn and Quinn (2010) have shown AIPW’s superiority over other treatment effect methods. One important advantage of the AIPW technique is that it is “doubly robust” — statisticians have shown that if either the treatment model or the outcome model (but not both) is incorrectly specified then the method nevertheless yields unbiased estimates of the effect of the treatment (Funk, Westreich, Stürmer, Brookhart, & Davidian, 2011; Lunceford & Davidian, 2004; Tan, 2010).

## Findings

Table 2 reports relationships between credits and employment outcomes as simple averages without controlling for any other variables. The first row reports these outcomes for year 2000 graduates who did not attend college thereafter. The next several rows refer to students who attended college but did not graduate with a credential. These non-graduates are subdivided by how many credits they accumulated. The bottom rows provide earnings and employment data for students who did complete various credentials.

**Table 2.** Percent employed and mean earnings by educational attainment.

	% Employed	Mean Earnings
No college credits	35.2%	$37,675
1–12 Credits	50.5%	$43,732
13–60 Credits	54.6%	$40,315
61+ Credits	58.3%	$41,576
OSA	64.7%	$34,627
Short certificate	67.2%	$40,768
Long certificate	75.1%	$43,945
Academic associates	64.8%	$42,061
Applied associates	75.4%	$55,049
Bachelors of applied tech	71.8%	$47,266
Bachelors (BA/BS)	64.8%	$64,727
Graduate	70.9%	$74,421

There is a pattern: students who attend college short of a degree are much more likely to be employed than members of the cohort who did not go to college, and, if employed, they have higher earnings on average. An exception is the Occupational Skill Award (OSA). This very short-term credential is quite rare in this cohort (N = 312, or 0.2%). OSA awardees are more likely to be employed, but their earnings are lower, compared to non-college goers.

For those who attend college but don’t complete a credential, the likelihood of employment increases with greater numbers of credits accumulated, although college students with the lowest number of credits (1–12) had higher earnings than students who had earned more credits.

Table 3 demonstrates a similar relationship between credits or credentials and employment outcomes but now based on regression analyses with statistical controls for student socio-demographics, academic performance in high school, and high-school fixed-effects. Across both models, college non-completers are significantly more likely to be employed than students who forgo college by roughly twenty percentage points, and college attendance short of a credential was also positively related to earnings in most cases. For example, students who earned 31–60 credits at a community college received an earnings boost roughly half the magnitude of students who earned an academic associate’s degree (7.4% vs. 14.3%)Yet two unexpected findings emerged from this analysis. Attending a community college short of a degree was estimated to have a more pronounced impact on earnings compared to attending a public 4-year school short of a degree. Additionally, for 4-year students compared to non-college goers, only non-completing students with 1–12 credits received a statistically significant earnings benefit, whereas students with greater numbers of credits (but short of a degree) did not.

**Table 3.** OLS regression with controls and high school fixed effects.

	2-Year college entrants compared to no college		4-Year college entrants compared to no college	
	Employed	Log earnings	Employed	Log earnings
*Model 1*				
Any credits vs. No credits	0.198***	0.058***	0.205***	0.012
	(0.0032)	(0.0099)	(0.0054)	(0.0181)
*Model 2*				
Number of credits or highest credential category				
1–15 Credits	0.154***	0.0697***	0.105***	0.0998**
	(0.00468)	(0.0134)	(0.0141)	(0.0434)
16–30 Credits	0.179***	0.0395***	0.123***	0.03
	(0.00515)	(0.0143)	(0.0124)	(0.0363)
31–60 Credits	0.204***	0.0744***	0.175***	0.0195
	(0.00470)	(0.0131)	(0.0095)	(0.0272)
61–120 Credits	0.241***	0.0973***	0.229***	0.0449*
	(0.00485)	(0.0134)	(0.0082)	(0.0230)
121+ Credits	0.243***	0.0337*	0.275***	0.0351
	(0.00764)	(0.0203)	(0.0100)	(0.0273)
Short certificate	0.286***	0.179***	0.322***	0.110***
	(0.00711)	(0.0182)	(0.0150)	(0.0372)
Long certificate	0.377***	0.427***	0.481***	0.444***
	(0.0208)	(0.0504)	(0.0584)	(0.1380)
Applied associates	0.396***	0.474***	0.416***	0.520***
	(0.00744)	(0.0187)	(0.0196)	(0.0491)
Academic associates	0.294***	0.143***	0.334***	0.0746**
	(0.00685)	(0.0180)	(0.0141)	(0.0357)
Bachelors of applied tech	0.371***	0.302***	0.380***	0.216***
	(0.0192)	(0.0460)	(0.0286)	(0.0696)
Bachelors	0.351***	0.461***	0.346***	0.465***
	(0.00405)	(0.0117)	(0.0047)	(0.0146)
Graduate	0.438***	0.620***	0.435***	0.634***
	(0.00674)	(0.0179)	(0.0074)	(0.0207)
N	102,091	56,732	64,272	30,835

Table 4 provides a different set of analyses of the employment outcomes, contrasting no-college with some college groups, using the AIPW method that reduces selection bias. The leftmost column reports the treatment effect comparing all non-college goers with all “some college” (short of a credential) cases. The remaining columns provide AIPW estimates for distinct subpopulations: (1) non-economically disadvantaged students only, (2) economically disadvantaged students only, (3) men only, (4) women only, (5) Black only, (6) Hispanic only, (7) non-Hispanic whites only.

**Table 4.** AIPW treatment models on full employment, comparing those who never attended college to “some college” or non-completers.

	All	Non-disadvantaged	Economic disadvantaged	Male	Female	Black	Hispanic	White
2-Year college non-completers								
Treatment effect	.201***	.195***	.211***	.187***	.215***	.238***	.225***	.165***
Standard error	.0032	.0039	.0058	.0045	.0046	.0087	.0054	.0047
N	102,091	68,456	33,635	54,215	47,876	14,032	38,213	47,449
4-year college non-completers								
Treatment effect	.188***	.171***	.221***	.168***	.215***	.203***	.216***	.158***
Standard error	.0075	.0094	.0187	.0126	.0119	.0155	.0186	.0135
N	64,272	41,467	22,805	35,608	28,664	9,769	24,775	28,015

In the top panel of Table 4—for community college entrants—there is a statistically significant and substantively large increase in the percent “fully employed”, when comparing those with some college and no credential with cohort members who never went to college. The effect of some college on employment is significant for the cohort as a whole: there was a 20.1 percentage point difference in employment between those with some college and those with no college. A similarly large employment advantage was apparent for the gender and ethnic subpopulations, and for those students who were classified as economically disadvantaged during high school. All were statistically significant and 16 percentage points or larger.

The bottom panel of Table 4 provides equivalent analyses comparing no college with those who entered a four-year college but did not complete a credential. The treatment effects for the group as a whole and for the subgroups are all statistically significant and large: the employment gap between no college and some college short of a degree was 18.8 percentage points for the group as a whole, and estimates of the treatment effect for each subgroup model were at least 15 percentage points and statistically significant.

Table 5 reports treatment effects for the log annual wages outcome. Note that this analysis is limited to persons who had non-zero wages in 2015. It therefore represents the effect of some college, contingent on having earnings. Consequently, the sample sizes are smaller than in the prior table.

**Table 5.** AIPW treatment models on post-college earnings (logged) comparing those who never attended college to others with “some college” or non-completers.

	All	Non-disadvantaged	Economic disadvantaged	Male	Female	Black	Hispanic	White
2-Year college non-completers								
Treatment effect	.065***	.056***	.081***	.026**	.114***	.068***	.045***	.074***
Standard error	.0010	.0127	.0170	.0133	.0156	.0276	.0146	.0161
N	56,732	37,512	19,220	31,475	25,257	8,086	22,227	25,578
4-year college non-completers								
Treatment effect	.058***	.024	.226***	.026	.132***	.005	.0769	.043
Standard error	.0341	.0901	.0901	.0608	.0370	.0620	.0492	.0723
N	30,835	19,460	11,375	18,236	12,599	4,866	12,318	13,195

There are interesting commonalities and divergences between the earnings results for the 2-year and 4-year samples. The treatment effect estimates for the overall samples are quite similar: community college “some college” students receive a 6.5% earnings boost, and public 4-year some college students receive a 5.8% increase in earnings compared to students who never attend college. Similarly, among enrollees in either sector, women non-completers gain far more earnings benefit from college attendance than men. Males receive a 2.6% earnings advantage for attending a 2-year or 4-year college without completing, compared to 11.4% and 13.2% increases in earnings for women. Economically-disadvantaged students also receive larger earnings benefits from incomplete college compared to non-disadvantaged students.

But there are some noticeable differences as well. Although economically disadvantaged students do receive a greater benefit from incomplete 2-year college enrollment compared to non-disadvantaged, the difference is only 2.5%. In contrast, non-disadvantaged students who attend 4-year colleges short of a credential receive almost no increase in earnings, whereas disadvantaged non-completing students receive a 22.6% increase in earnings, the largest effect estimate for any subgroup earnings model. Another key difference is that none of the treatment effect estimates on earnings were statistically significant for the racial/ethnic subgroup models for 4-year enrollees, yet college enrollment short of a credential was estimated to significantly increase earnings for all subgroups in the 2-year enrollment models. This is likely partly due to sample size differences, as the samples for 2-year enrollees are roughly twice as large as the 4-year models, as well as the possibility that there is greater variation in earnings (and thus error in the estimates) among 4-year college attendees.

### Robustness checks

To check whether our findings were sensitive to the particular ways we constructed analytical models, we carried out alternative specifications: we defined the earnings measures as dollar earnings, and as logged earnings as well as top-coded and then logged earnings. We examined employment in any quarter of 2015, in no quarter, or in every quarter of the year. We modeled the contrast of no college compared to some college using conventional regression models, with fixed effects regression models controlling for the high school that students enrolled in, with augmented inverse probability weights, and using an alternative counterfactual method: IPW with regression adjustment. We also ran earnings models restricting the sample to students who were employed full-time to disentangle the potential effect of wage increases from increases in the amount of work obtained. The results were generally quite consistent across all these specifications, particularly for 2-year college enrollees: individuals with some college short of a credential have better employment and earnings levels, on average, than those who do not go beyond high school, after controls for socio-demographic background and academic performance in high school. The estimates of the impact of some college-going on employment were also quite consistent across models for 4-year entrants. These results regarding robustness checks are available upon request to the corresponding author.

## Limitations

The Texas TERC dataset tracks many millions of students from K-12 schooling into post-secondary education and into the labor force. However, its coverage is not universal because approximately 5% of Texas high school graduates enroll in college out of state, and are therefore lost to the TERC tracking database, compared to the roughly 50% of Texas high school graduates who attend college within Texas and the remainder of the cohort do not enter higher education, all of whom are in the TERC database. Any undergraduates who leave Texas for college and then return to Texas and are employed in Texas will appear in the TERC database to be in the “no college” group—a misclassification.

Although a small percentage of the cohort, this would produce a *conservative bias*: some persons who received either a partial or a complete college education out-of-state and who likely earn more on average than their high school graduate counterparts will be classified as having “no college” because TERC has no record of their out-of-state higher education. These misclassified cases would likely reduce the earnings differential between “no college” and “some college” groups. Our finding is that there are substantial and statistically significant differences in employment and earnings between these two groups, despite the fact that these types of misclassified cases would reduce the observed difference below what it would otherwise have been. Our results therefore provide an understatement of the employment benefits of having some college compared to no college.

## Discussion

The analyses presented here document substantial employment benefits from attending either a two-year or a four-year college for those students who do not complete a degree or credential, congruent with a number of prior studies (Jacobson et al., 2005; Jepsen et al., 2014; Kane & Rouse, 1995, 1999; Marcotte et al., 2005). The relationship between some college and employment is especially strong. This is the first payoff to college even for those who do not complete a credential. Students who do not go beyond high school are considerably less likely to be employed fifteen years later than their “some college” counterparts, even after controlling for their academic preparation and socio-demographic characteristics.

A second advantage of some college was observed among those high school graduates who were employed and earning years later. Those with some college had significantly higher earnings years later than their contemporaries who never attended college. Very similar findings were discovered when using conventional regression models and when using modeling techniques that address selection bias, and when alternative specifications of variables were employed: they seem quite robust.

Our findings add to the evidence base suggesting that college attendance may provide valuable socioeconomic benefits to students even if they do not complete a course of study. Clearly it is better for undergraduates to earn a credential, and that should be the goal for all undergraduates and colleges alike. But the many students who attend college but do not make it through to a credential are not wasting their time: their employability prospects and their earnings years after leaving college are higher on average than those of similar graduates who never went beyond high school. Attending college short of a degree is a substantial stepping-stone toward a better living for the average non-completer, and this finding also applies to those from minority groups and economically disadvantaged backgrounds who enter community colleges but do not complete a credential there.

This finding is of particular importance to state and institutional policy given the growth of state policy approaches aimed at increasing degree attainment (HCM Strategists, 2016), particularly outcomes-based funding approaches (Friedel et al., 2013; Jones, 2014; National Conference of State Legislatures, 2014; Tandberg & Hillman, 2014). College presidents and administrators often report that declines in state appropriations for higher education require them to increase tuition and fees, potentially limiting access to students who cannot afford the higher costs (Immerwahr, Johnson, & Gasbarra, 2008). Many college ranking systems (e.g., US News and World Reports) also privilege selectivity in rankings, both in terms of the percentage of students admitted/rejected and the standardized test scores of the student body. Combined with outcomes-based funding, lack of sufficient resources to support student success may create a strong incentive to admit only those students who are more likely to complete a degree and, conversely, exclude students with lower estimates of completion. Indeed, both qualitative (Dougherty et al., 2014) and quantitative (Umbricht, Fernandez, & Ortagus, 2017) research suggests this may be precisely how institutions are responding to outcomes-based approaches.

These findings notwithstanding, an important caveat is that we did not directly test whether the labor benefits of college non-completion are a result of the human capital students accumulated, the positive signal provided by college attendance, or a more nefarious alternative such as students falsely claiming that they completed a credential (Attewell & Domina, 2011). Unfortunately, during the time period under study, Texas did not collect data on the specific courses students attempted and completed, preventing us from examining whether the return to credits varied by subject or field. Prior literature suggests the returns do vary by field (Bahr, 2016; Grubb, 1992, 1993), which may be more congruent with the human capital approach. However, the fact that students with the lowest number of credits often received the greatest earnings benefit (though not in terms of employment) may suggest that the simple act of enrolling in college is a strong signal to the labor market, even before students have accumulated significantly more human capital.

It is also important to reiterate the fact that signals are highly contextual, making the benefits of college non-completion proportional to the supply and demand of workers in a particular labor market. This might explain why the benefits of community college attendance were estimated to outweigh the benefits of 4-year attendance in some cases. If students who attend 4-year colleges seek work in a more competitive labor market the signal of being a “college dropout” may be more negative, resulting in less employment benefits compared to non-college-goers.

Regardless of whether the benefits of college attendance are driven by human capital or by signaling effects, our results suggest that students who attend college short of a degree have better economic outcomes than their observably equivalent peers on average. Combined with our finding, and the growing literature base, suggesting that students with the lowest likelihood of college-going often receive the greatest benefits from college (Brand & Xie, 2010; Card, 2001; Heckman & Li, 2003), our results imply that excluding students from higher education might do greater harm than benefit to both students and society, even if admitted students are not very likely to graduate. Similarly, our results oppose the notion that college non-completers have simply wasted their time and resources, as well as the resources of the public sector (Holzer & Baum, 2017; Rosenbaum et al., 2015). Numerically, the some-college group is enormous and should not be viewed as a residual category but should be studied in its own right. For many non-completers, attending college is a useful stepping-stone into the labor market.

## Notes

1. Calculated from Shapiro et al. (2017) Appendix B, Tables 14 & 20.

2. Of course, the impact of a college education goes well beyond employment and earnings outcomes. See Trostel (2015), Lagemann and Lewis (2012), and Pascarella and Terenzini (2005) for the broader benefits of college attendance.

3. FUTA requires the collection of wage data for the vast majority of workers, but not all. Categories of employment that are generally not covered by states’ UI wage systems include: (1) self-employment, (2) certain agricultural labor and domestic service, (3) service for relatives, (4) service of patients in hospitals, (5) certain student interns, (6) certain alien farm workers, (7) certain seasonal camp workers, and (8) railroad workers (Feldbaum & Harmon, n.d., p. 6). Despite these exclusions, an estimated 99.7% of all wage and salary workers and 89% of the civilian labor force in the US are covered by UI wage collection (Feldbaum & Harmon, n.d., p. 7). Members of the military are also excluded from UI data systems nationwide.

## Appendix 1

**Table:**

	N	Min	Max	Mean	SD
Female	207,332	0.00	1.00	0.51	0.50
Amer Ind	207,332	0.00	1.00	0.00	0.05
Asian	207,332	0.00	1.00	0.03	0.18
Black	207,332	0.00	1.00	0.13	0.33
Hispanic	207,332	0.00	1.00	0.32	0.47
White	207,332	0.00	1.00	0.52	0.50
Econ Dis	207,332	0.00	1.00	0.27	0.44
Gifted	207,332	0.00	1.00	0.13	0.33
Math Assessment	195,636	1.00	99.00	67.83	25.34
Reading Assessment	195,636	1.00	99.00	63.57	27.47
HS Credits	207,332	0.00	61.00	25.20	6.54
HS Advanced Credits	207,332	0.00	16.00	1.51	2.24
HS Dual Credits	207,332	0.00	10.00	0.18	0.66
College Credits	207,332	0.00	665.00	69.29	74.22
Any College Credits	207,332	0.00	1.00	0.69	0.46
Non-Employed	207,332	0.00	1.00	0.37	0.48
Full Employment	207,332	0.00	1.00	0.54	0.50
Earnings	131,561	0.01	6,716,352.76	50,769.63	63,427.34
Earnings (Top-Coded)	131,561	0.01	214,536.15	49,385.03	38,207.44
Log-Earnings	131,561	−4.61	15.72	10.42	1.13
Log-Earnings (Top-Coded)	131,561	−4.61	12.28	10.42	1.13
